# Dynamic variance-aware federated tuning for efficient autonomous vehicle perception under non-IID settings

**DOI:** 10.3389/frobt.2026.1824246

**Published:** 2026-06-17

**Authors:** V. Dhanavarshini, Sasikumar Periyasamy

**Affiliations:** School of Electronics Engineering, Vellore Institute of Technology, Vellore, Tamilnadu, India

**Keywords:** autonomous vehicle (AV), dynamic variance-aware federated tuning, Federated Learning (FL), non-IID dataset, object detection (OD)

## Abstract

**Introduction:**

Federated learning enables multiple autonomous vehicles (AVs) to collaboratively train machine learning models while preserving data privacy. However, performance degrades significantly under non-independent and identically distributed (non-IID) data conditions commonly encountered in real-world driving scenarios. Existing aggregation methods, particularly Federated Averaging (FedAvg), struggle to effectively handle client update divergence, leading to inefficient communication, unstable convergence, and increased privacy risks.

**Methods:**

To address these challenges, we propose a Dynamic Variance-Aware Federated Tuning (DV-FedTune) framework for object detection in autonomous driving systems using YOLOv12. The proposed framework dynamically adjusts client contributions through a variance-aware aggregation strategy that jointly models update consistency, variance-based diversity, and loss-guided reliability using a round-adaptive weighting mechanism.

**Results:**

Comprehensive experiments were conducted on the KITTI object detection dataset under various non-IID federated learning configurations involving different numbers of clients, local training durations, and communication rounds. The results demonstrate that DV-FedTune consistently outperforms FedAvg, Exponential Moving Average in Federated Learning (EWHFed), and VINOEffiFedAV in terms of communication efficiency, computational cost, and model performance while maintaining stronger privacy preservation.

**Discussion:**

The proposed framework achieves stable aggregation behavior and effective parameter utilization as the federated network scales. These findings indicate that DV-FedTune provides an efficient and privacy-preserving federated learning solution for distributed object detection in autonomous vehicle environments operating under heterogeneous data distributions.

## 1 Introduction

Robust perception systems are relied upon heavily by AVs to ensure safe and reliable operation in complex and dynamic traffic environments. Among the various perception tasks, real-time road object detection is fundamental for scene understanding, collision avoidance, and motion planning. Recent advances in deep learning have positioned single-stage object detectors, particularly YOLO-based architectures, as strong candidates for autonomous driving due to their favourable balance between accuracy and computational efficiency (Jiang, 2025). To further improve detection reliability in real-world driving conditions, localization accuracy and robustness have been focused on enhancing through architectural and sensor-level innovations by several studies. Improved depth perception and spatial consistency approaches integrate binocular vision with optimized YOLO models, enabling more precise detection of road objects under challenging conditions such as occlusion and scale variation (Mohammadi and Ferdowsi, 2025). Complementary to architectural improvements, attention-based and risk-driven detection strategies emphasize safety-critical regions in the driving scene, thereby enhancing perception reliability in dense traffic scenarios (Wolfe et al., 2024). Adaptive perception pipelines gain attention as they dynamically adjust object detection based on driving context and environmental complexity. Sequential and adaptive detection strategies enable relevant objects to be prioritized while maintaining real-time performance, which is crucial for on-board deployment (Rawlley et al., 2025). Researchers have explored cross-domain modeling techniques in parallel to address domain shifts caused by varying weather, lighting, and geographic conditions, significantly improving generalization capability in dynamic driving environments (Wang et al., 2021). Beyond perception accuracy, decision-making and system-level integration play a vital role in autonomous driving. Hierarchical decision-making frameworks have been proposed to couple perception outputs with vehicle control and behavioural planning, allowing complex driving scenarios to be handled more effectively by AVs (Yuan et al., 2024). Additionally, adaptability and trust have been shown to improve through the incorporation of human feedback into autonomous systems, aligning machine perception and decision-making with human driving behaviour (Cui et al., 2025).

A key challenge associated with the growing use of connected autonomous cars is data privacy and transmission efficacy; these issues can be resolved through FL (FL) that allows collaborative training of models without the need for transfer of raw sensor data from individual vehicles (Wang et al., 2025). Nevertheless, FL faces some major challenges related to heterogeneous client data distributions and unstable convergence due to client drift in the application of autonomous driving. There have been numerous adaptations of FL to address these challenges by employing fairness-aware and loss-weighted aggregation approaches that focus on providing a balance of contribution from clients participating in aggregate model construction across different non-IID data distributions, thereby making a more stable model globally (Ali et al., 2025). Another approach to enhance performance in FL was through applying reinforcement learning-based optimization techniques for evaluating and optimizing the complete autonomous driving pipeline through simulation tested environments indicating a need for dynamic optimization technique development for learning-based autonomous driving systems (Cai et al., 2025). Studies have demonstrated that deep learning models (e.g., neural networks) can be highly sensitive with respect to the differences observed in non-IID data sets, thus providing further justification for developing reliable aggregation strategies when applying FL systems for AVs driving applications (Behera et al., 2025). The performance of FL has also been focused on enhancing in terms of convergence and robustness via adaptive optimization, clustering, and semi-decentralized frameworks by recent research. Techniques such as adaptive local aggregation, clustered FL, and incremental optimization, have shown promise in addressing the issue of performance degradation due to heterogeneous client datasets and uneven participation due to their heterogeneity (Devarajan et al., 2025). However, existing methods tend to focus exclusively on privacy preservation or fairness or communication efficiency, making it difficult for them to perform well in a complex, autonomous driving environment. To tackle these issues, this paper presents DV-FedTune, a dynamic, variance-aware federated tuning approach for object detection in AVs. DV-FedTune is different from traditional FedAvg methods and more recent methods that focus on efficiency since it takes into account the diversity of the client updates as well as the dynamics of the client loss during training rounds and how reliably those updates are aggregated. We demonstrate through extensive experimentation using the KITTI dataset that DV-FedTune outperforms baselines such as FedAvg, EWHFed, and VINO_EffiFedAV with respect to detection accuracy, convergence stability, and overall communication and computational efficiency. Ultimately, these results demonstrate that DV-FedTune offers an effective and scalable option for privacy-preserving, high-performance perception in AVs.

## 2 Related works

### 2.1 Object detection models for autonomous driving (YOLO-based)

Real-time object detection is important in the autonomy of driving systems; both accurate location and fast detection play a role as input into critical decision making in terms of safety. Although there are several one-stage detectors available, one of the leading approaches currently being used is the YOLO architecture because of its low inference time and decent accuracy in complex driving environments. As well, there are many problems with the YOLO architecture in terms of its ability to detect small objects, different sizes, occlusion, and adverse weather conditions, which all limit its ability to operate effectively in real-world autonomous driving scenarios. Enhanced architectures of YOLO designed for object detection within roadway environments have been developed to try to enhance performance when detecting objects in high-density traffic scenes. Using a YOLOv8-based framework has improved detection capabilities by incorporating optimized features into a backbone module for feature extraction and improving feature-level fusion either through added attention mechanism capabilities or revised loss functions for bounding box regression when detecting both distant or overlapped vehicles as well as vulnerable road users, resulting in improved accuracy on benchmark tests (Gao et al., 2025). The lightweight design for models has lately been one of the major research areas to help facilitate deployable embedded platforms in an automotive environment. An example is the Pre-convolution Receptive Field Enhancement and Dynamically Reconfigurable YOLO (PCPE-YOLO), which proposes a dynamically reconfigurable backbone architecture combined with efficient contextual feature extraction in order to achieve a balance between computational efficiency and object detection accuracy. In addition, this model introduces dedicated small object detection mechanisms and significantly reduces the total parameter count, suggesting great suitability to edge-controlled autonomous driving (Chen et al., 2025). Several studies beyond traditional vehicle-based visual detection with YOLO have investigated its applications to complex and heterogeneous environments, including roadside ecological scenes and in multi-domain environments. From the results of comparative analysis across YOLO architectures where particular layers resemble the functionality of the transformer architecture, it was shown that the inclusion of those components improves contextual understanding and provides additional robustness to scale variations; however, the trade-off is higher computational complexity (Raza et al., 2025). The results of those studies illustrate the need for careful trade-offs regarding real-time constraints and accuracy as it pertains to autonomous systems. Traffic sign detection is a challenge due to large variability of size, perceptual degradation, and physical interference from the surrounding environment. Cascade feature and recursive fusion techniques have been proposed to preserve shallow spatial features while concurrently retaining deep semantic representations, which resulted in improved detection rates for small and perceptually degraded traffic signs (Liu et al., 2025). These newest developments consist of applying multiscale attention systems and improving the performance of previously developed systems by integrating weather measures (using other methods). Compared to previous versions, these recently created systems have improved capabilities in handling unfavourable weather circumstances including rain, fog, or poor illumination (Li J. et al., 2025). Research shows that when YOLO systems operate independently, there is a favourable trend in accuracy, robustness, and efficacy. However, issues with generalisability and scalability in many contexts still need a lot of attention in current research.

### 2.2 FL fundamentals and non-IID challenges

A decentralized approach called FL allows many clients to work together to build a worldwide model without having to give out their raw data. This means there are fewer concerns with privacy, security, or ownership of data that happen with centralised methods. The main algorithm used in FL is FedAvg. It combines updates that clients made to their local models into a single global model by using a central server. Many different applications have used FL due to the success of FedAvg. The big issue with FL when it is put into practice is that the performance is usually greatly reduced by the clients’ non-IID. The lack of similarity between what each client is using causes convergence to occur slowly, global models to be biased, and much more communications to occur. To improve the privacy aspect of FL, various forms of differentially private FedAvg (i.e., adding calibrated noise to the updates) have been created. These implementations offer formal privacy protections to the client while at the same time provide assurance that they will converge even when there are only some clients and their data are non-IID, which makes them a baseline for privacy-friendly FL systems (Zhang L. et al., 2025). However, adding noise introduces even more challenges to optimisation in heterogeneous federations. The different characteristics of non-IID data, such as inadequate labels or size imbalances of families or groupings of data, have been studied methodically across various usage domains, such as data sentiment analysis. According to empirical studies, the skew of the label distribution has a more significant, detrimental influence on the performance of the model than the imbalance of the data due to incomplete data, while more capable architectures (i.e., transformer-based) were able to demonstrate better robustness when confronted with this phenomenon. Thus, there is a need for FL algorithms that can adaptively address the heterogeneous nature of these statistical data effects. Many researchers have proposed algorithmic modifications to alleviate the negative effects of non-IID data through adaptive optimization and sampling methods. To simultaneously mitigate privacy leakage and convergence instability due to non-IID settings, the authors of several studies (Gholamiangonabadi and Grolinger, 2024) developed non-IID FL algorithms based on differential privacy (DP), incorporating truncated concentration bounds and unbiased client sampling within the algorithm to support improved performance compared to standard DP-FedAvg in heterogeneous data settings (Chen et al., 2024). A complementary group of studies focused on developing entropy-based data quality evaluation mechanisms like, FedAvg-BE to provide selective emphasis on informative local updates, thereby reducing bias and accelerating convergence speed in non-IID settings (Orlandi et al., 2023). To manage client drift introduced by local training, researchers have examined alternatives to weight normalisation or standardization techniques like Federated Learning with Weight Standardization (FedWS) to improve model accuracy (by reducing the divergence of gradients between clients) and lower communication costs in heterogeneous mobile and Internet of things (IoT) environments (Vieira and Campos, 2025). In addition to the challenges of heterogeneity associated with static data sources, recent studies also document the impact of combined spatial and temporal variations in the data and, in particular, that the underlying mechanisms for client drift and catastrophic forgetting are both linked to changes in the underlying distributions. The use of unified analytical frameworks also indicates that there is more complex behaviour that cannot be attributed solely to random effects and there are specific circumstances where there can be a significant performance gain due to moderate non-IID shifts (Babendererde et al., 2025). In general, the findings of the studies demonstrate that the identification and management of non-IID data is still an important area of research for solving the challenges facing FL and the continued development of sophisticated aggregation, normalization and adaptive learning techniques specifically for use in heterogeneous environments.

### 2.3 Advanced aggregation and optimization in FL

The simple FedAvg aggregation method is a well-established, scalable method for aggregating information and performing a straightforward and practical form of optimization using the decentralized structure of FL. However, the inadequate performance of FedAvg in heterogeneous environments with non-IID distributions has led to significant efforts in exploring alternative aggregation and optimization approaches. Traditional static averaging approaches do not adequately capture the variability of data quality across clients, the reliability of client updates, and the heterogeneity of the overall federated system, all of which contribute to unstable convergence and result in global models that are biased when deploying FL in practice. As a result, adaptive aggregation approaches have recently been proposed to address the limitations of aggregation in heterogeneous environments. A prime example of an adaptive aggregation approach is Federated Self-Adaptive Learning for Information Networks (FedSIN). A self-adaptive aggregation technique that incorporates an information network model to capture inter-client dependency relationships is employed by FedSIN. Additionally, FedSIN enhances convergence stability and improves representation learning performance for non-IID data distributions by adaptively computing the aggregation weights for each client based on their inferred relevance (Li A. et al., 2025). Similarly, fair FL approaches have been proposed that utilise an exponential moving average for weighting client loss to balance the contribution of each client, thus minimizing the influence of data-rich clients while improving the overall fairness and robustness of the federated model (Mollanejad et al., 2025). In addition to traditional client-driven aggregation methods, interest in recent research has also focused on cloud computing data-free and knowledge distillation-based approaches to optimization. Specifically, diffusion-based generative methods have been proposed that allow the refinement of global FL models through the generation of synthetic representations of client data, eliminating the need for access to the private data of individual clients while mitigating the effects of unevenness introduced by non-IIDs. The effectiveness of global FL models is significantly improved by these methods while the communication overhead associated with aggregating clients is reduced, especially in edge environments that are resource-constrained (Najafi et al., 2024). Hierarchical Aggregation Techniques improve scalability through client organization into systematized groups. Semi-decentralized frameworks for FL commonly utilize incremental approaches to sub-gradient optimization within clusters, thereby benefiting from minimized communication costs as well as reducing client drift within large scale federated systems (Huang et al., 2025). The Client Clustering paradigm has proven effective in addressing extreme levels of heterogeneity. Label wise clustering techniques utilize the similarity of class distributions as an organizational basis among clients, allowing for a more coherent agglomeration of clients in order to produce improved rates of convergence under scenarios of non-IID between classes or data sources (Lee and Seo, 2023). Inference Driven Clustering techniques expand this concept by using the nature of clients’ prediction behavior as a basis for cluster formation vs. the statistics of the raw data, thereby allowing for improved functional similarity across models and consequently better agglomeration processes among clients (Morafah et al., 2023). Robust aggregation under adversarial conditions has also been explored using client-versioning with Aggregation Filtering (i.e., two stage optimization) techniques such as Federated Client Verification and Gradient memory (FedCVG), where client filtering and virtual agglomeration are complentary strategies used to mitigate against the impacting effects of incorrect or malicious updates being sent from clients to the coordinator/server (Zhang R. et al., 2025). Optimization oriented toward efficiency has also benefited aggregated computations performed among federated clients. Efficient Federated Learning with Optimal Local Epochs (FedEff) dynamically determines the optimal number of local epochs for training each client using a dual assessment function to measure trade-offs between computational performance and communication requirements resulting in an overall improved rate of convergence for heterogeneous systems (Narmadha and Varalakshmi, 2025). Normalization based complimentary solutions were also proposed as a means for reducing client divergence of weights by standardizing local updates before their aggregation will continue to lead to better stability and less drift for all models working with many different types of data from a variety sources (Domini et al., 2026). Advanced aggregation and optimization strategies suggest that adaptive weighting, clustering, normalization and efficiency-aware mechanisms are necessary for successful FL. But many current solutions tackle these problems separately. Therefore, it is important to develop comprehensive frameworks that integrate considerations about update reliability, data heterogeneity and system efficiency.

### 2.4 FL for AVs and security

FL has become an important technology for AVs as it provides a way to cooperate with other vehicles to train their models while keeping their data private. However, there are a number of challenges that must be addressed before FL can be used in an AV ecosystem including non-IID data from the fleet of vehicles, unbalanced label distributions, limited communication resources and security threats from malicious actors. A significant amount of research has been done in the last several years on how to address these issues. One recent works, “Federated Virtual Clients (FedVC): A Peer-to-Peer FL Framework for Balanced and Heterogeneous Data in Autonomous Driving”, proposes a peer-to-peer FL framework that helps to achieve better approximations of global data distributions by using virtual clients for each physical vehicle to represent larger groups of physical vehicles as they consume local data for learning model weights (Zhang Y. et al., 2025). The authors demonstrate that by controlling sampling of local data segments and timing of backpropagation, their approach improves collaborative learning while maintaining data privacy. The authors also show that it outperforms both FedAvg and other baseline approaches on several different AV data sets that have varying degrees of label imbalance. The work Federated Region-learning framework of personalized Autonomous Vehicles (FedRAV), is based on the principles of personalization and studies the use of hypernetworks in order to create personalized regional and vehicular models by splitting large geographic areas into smaller regional areas and aggregating the models created for each vehicle using nearby vehicle data by using an adaptive aggregation strategy. The authors show that this adaptive regional aggregation approach improves model accuracy across all three AV data sets under non-IID conditions (Zhai et al., 2024). Besides improving performance, federated autonomous driving systems require increased emphasis on fairness and enhanced security. Establishing a FL framework that is fair and secure will involve developing a process for assigning personalized local training rounds based on trade-offs between balanced energy consumption and convergence speed. Mechanisms for detecting attacks will be incorporated into this framework and will include Gini impurity to identify and mitigate malicious users. Experimental results indicate greater energy fairness, shorter training times and a higher degree of robustness against adversarial behaviour (Fu et al., 2024). Privacy solutions that preserve the original source of exposure to data and mitigate processing overhead concerns are referred to as complementary solutions. An example of a complementary solution for privacy protection is Gradient Encryption based FL (“GeFL”). GeFL allows collaborative training of data across federated workers without adding extra communication overhead or computation. Further, it provides the ability to have improved accuracy of results while at the same time reducing the volume of data being transferred between workers in a cooperative driving task involving detecting traffic signs (Parekh et al., 2023). In addition to privacy concerns, cybersecurity presents another challenge for autonomous driving systems that are designed to work together as a group, or cooperatively connected. The federated agents on vehicle platooning (FedAV) will provide researchers and practitioners with a method for using Federated Anomaly Detection techniques and approaches to conduct research on various types of cyberattacks, including spoofing and replay attacks against a collection of vehicles operating together as part of a vehicle platoon (Lin et al., 2025). Finally, research on benchmarking model poisoning attacks against Byzantine resistant pooling algorithms will allow researchers to better understand the extent of the security vulnerabilities, robustness of the algorithms against these attacks, and effectiveness of the algorithms under both IID and non-IID conditions. These studies indicate that selecting strategic clients may be a good way for traditional beliefs about data distribution and security in FL to be countered by FL (Almutairi and Barnawi, 2024) researchers as an effective deterrent to sophisticated attacks. While identifying targeted defense mechanisms has been the focus of many researchers in exploring possible countermeasures to sophisticated attacks, they continue to do so through several methods of gradient-based clustering in which malicious updates from multi-label flipping attacks were filtered and identified and the overall performance was enhanced regardless of the various datasets/attack scenarios used (Ma et al., 2025). In addition to providing security and robustness, there are also new applications of FL for more advanced perception styles including monocular depth estimation. Implementation of the Bayesian optimization strategies with FL have achieved large reductions in test loss and overall communications costs while providing a feasible deployment option for AVs (Soares et al., 2025). Finally, the VINO_EffiFedAV provides an efficiency-based FL framework for autonomous vehicle object detection by integrating selective updates from clients with YOLO-based models to reduce overall communication and computational costs but still maintain a strong edge when detecting objects in a non-IID data environment (Vinoth and Sasikumar, 2025).

## 3 Proposed work

**FIGURE 1:**
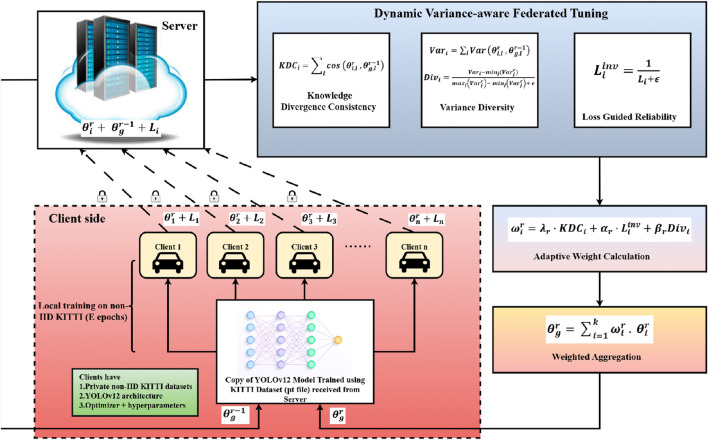
FL using DV-FedTune Aggregation.

FL for self-driving cars is challenging due to a lack of non-IID data distributions, inconsistent client updates and limits on the efficiency of large networks of vehicles. Each self-driving car logs unique traffic patterns (due the geography, time and weather) and will produce a vastly different, heterogeneous local dataset. The method of aggregation, FedAvg, treats all clients equally; therefore, because the global model is also based on the performance of low-performing clients, the global model will converge slowly and drop in accuracy when there are significant differences between the data distributions from the different clients. Aggregation methods based purely on efficiency and therefore only aggregate a subset of the clients do not typically evaluate the consistency and quality of the clients’ local updates. In order to solve these issues, this work proposes a new aggregation method called DV-FedTune for federated object detection in AVs shown in Figure 1. Since in these practical settings where decentralized perceptions are taking place, client datasets can vary greatly in terms of number of samples they contain, class makeup, and visual features. This variation causes current aggregation methods to produce unreliable convergence behaviors as well as lower performance. As part of our work, we implemented the existing FL methods of FedAvg, EWHFed, and VINO_EffiFedAV as our baselines and comparing their respective performances to that of our DV-FedTune approach. Rather than assigning equal weights to all client updates like FedAvg does or blending the global model with the current averaged update at the server side like EWHFed or using a static magnitude filter as Vino_EffiFedAv does, DV-FedTune provides a method whereby the client contributions to the aggregation are adjusted in a round-based manner based on how closely-their updates align with one another, how diverse they are from each other statistically, and how well they perform locally. The global model parameters will be represented by $\theta_{g}^{r}$ , which indicates the model’s parameters during federated round r. The locally optimized parameters from client i will be indicated by $\theta_{i}^{r}$ . Here θ is the set of learnable-weight parameters includes all the network parameter weights (i.e., weight parameters/sets of weights for the YOLOv12 convolutional layers, normalization layers and detection layers). Each client independently trains on the same non-IID partition of the KITTI dataset using the YOLOv12 model (E local epochs) by performing local optimizations after all training epochs per client. After all local client optimizations, each local client’s model will be evaluated by three criteria. The first type of evaluation will be performed by calculating the Knowledge Divergence Consistency (KDC) of client i according to Equation 1.

$$KDC_{i}=\underset{l}{\sum }\cos\left(\theta_{i,l}^{r},\theta_{g,l}^{r-1}\right)$$ (1)

At each layer l , the directional alignment of local and global parameters is measured. Disruptive updates will be withheld if they deviate too much from the lessons learnt from global data. The directional alignment of the client update relative to the global model is shown by the $KDC_{i}$ curve as the federated rounds progress. A KDC value that is high at the beginning indicates a stable agreement from the pretrained layer; the eventual decrease of KDC demonstrates how controlled client specialization occurs when clients are working with data that is not IID. The lack of major spikes in update variance indicates that DV-FedTune has the ability to suppress harmful divergence and maintain informative functional diversity. Further, the calculation of variance update

$$Var_{i}=\underset{l}{\sum }Var\left(\theta_{i,l}^{r},\theta_{g,l}^{r-1}\right)$$ (2)

The Statistical Dispersion of parameter update across layer l is measured using Equation 2, which captures the update heterogeneity caused by non-IID local data. The normalized diversity score is calculated using variance update, which is given in Equation 3

$$Div_{i}=\frac{Var_{i}-\min_{j}\left(Var_{j}^{r}\right)}{\max_{j}\left(Var_{j}^{r}\right)-\min_{j}\left(Var_{j}^{r}\right)+\epsilon }$$ (3)

where as j indicates all participating clients in round r , A small constant ϵ is used avoid division by zero, The smallest and largest variance among all clients $\min_{j}\left(Var_{j}^{r}\right)$ and $\max_{j}\left(Var_{j}^{r}\right)$ at round r This explicitly captures update diversity and prevents overemphasis on highly correlated client updates. Thirdly, loss-guided reliability Equation 4 is calculated using the inverse normalized validation loss $L_{i}^{\mathrm{inv}}$ . Each client evaluates its updated model on a local validation split after the local training to check the YOLO validation loss $L_{i}$ , which is transmitted to the server as a scalar reliability indicator along with the $\theta_{i}^{r}$ , local optimised parameter.

$$L_{i}^{\mathrm{inv}}=\frac{1}{L_{i}+\epsilon }$$ (4)

This prioritizes clients exhibiting better local generalization. Computing the Aggregation weights by combining these normalized metrics (such as KDC, Loss-guided reliability and normalised Diversity scores) using round-adaptive coefficients, Given in the Equation 5

$$\omega_{i}^{r}=\lambda_{r}\cdot KDC_{i}+\alpha_{r}\cdot L_{i}^{\mathrm{inv}}+\beta_{r}\cdot Div_{i}$$ (5)

where as $\alpha_{r}=\alpha_{0}e^{-\frac{r}{5}},\;\lambda_{r}=0.4\left(1-e^{-\frac{r}{5}}\right),\;\text{and}\;\beta_{r}=\beta_{0}+0.2\left(1-e^{-\frac{r}{7}}\right)$ The importance of this scheduling can be summarized as follows: higher $\lambda_{r}$ means less divergence due to extreme non-IID at the start of the rounds and a gradual increase of $\beta_{r}$ to support the gradual increase in informative diversity with continued training and the decreasing of $\alpha_{r}$ helps to mitigate overfitting at the end of the rounds since clients who are close to each other will have a similar local optimal solution. The ablation study shows that fixing these coefficients results in instability in convergence and that using adaptive scheduling produces a good balance between robustness and accuracy for convergence stability. $\theta_{g}^{r}$ is calculated as given in Equation 6.

$$\theta_{g}^{r}=\overset{k}{\underset{i=1}{\sum }}\omega_{i}^{r}\cdot \theta_{i}^{r}$$ (6)

The DV-FedTune aggregation approach overcomes the difficulties of non-IID data for YOLOv12 object detection by using a principled, adaptive federated aggregation approach that gives divergence-based consistency of knowledge alignment, variance-based diversity of information and loss-based reliability of updates during the process of aggregate client updates into a single global model. Unlike other static or single-dimensional aggregation approaches, DV-FedTune incorporates a round-adaptive schedule, which balances stability and diversity at various levels of the federated training process, thus allowing for controlled convergence in the initial rounds of training while providing for generalization in the last rounds of training. By using a round-adaptive schedule with the use of round-adjusted client aggregation in the round-adaptive aggregation process and client-specific exclusions of class-dependent detection head parameters, DV-FedTune minimizes label space inconsistencies that arise from decentralized detection tasks. Collectively, these design parameters provide for a stable, interpretable, and scalable aggregation process that mitigates harmful client drift while maintaining useful local knowledge during the aggregation of client updates. In this way, each client uses the pre-trained model to independently train on non-IID KITTI data while simultaneously aggregating client updates into the global model at runtime based on refinement based on divergence, variance, and loss through an adaptive weight scheme.

### 3.1 Dataset description

The experiments carried out with the KITTI Object Detection dataset with 7,464 images accessed through Roboflow, is a benchmarking dataset for the perception subsystem of autonomous driving, using high-resolution RGB cameras situated on vehicles and operating in real-world urban environments. The dataset contains annotated road scenes containing Truck, Van, Car, Pedestrian, Tram, Person_sitting, Miscellaneous and Car class objects, using tight bounding boxes as annotations. The images have been taken under a variety of lighting, scales for the object, occluded, and viewpoints; therefore, KITTI is well suited to test the real-world robustness of detection algorithms in addition to identifying how well they generalize to unseen data. Each image in the dataset was reformatted to fit into the YOLO annotation structure. Each image was then resized to 640 × 640. There are a total of 8 object classes across all experiments. The training dataset was randomly and unevenly partitioned across all of the clients at each training round to simulate non-IID FL; each client receives a non-static subset of samples for training. This random sample allocation ensures statistical heterogeneity, both in terms of class distributions and numbers of samples. Each client is assigned its own data.yaml file which contains references to the images and labels available to the client locally. Validation is also conducted locally by each client in order to compute client-specific losses which will be used for aggregating the scores of each client. These characteristics of the non-IID setup closely relate to decentralized perception systems of AVs, during which time each node is subject to the effects of a biased local environment.

**FIGURE 2:**
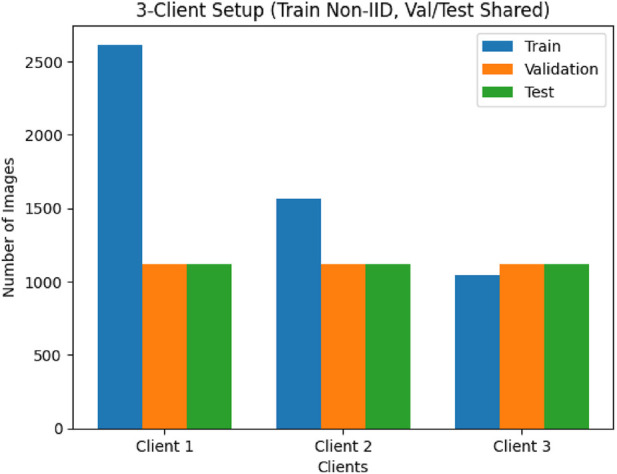
3 Client wise data split for federated Experiment (3 Clients).

**FIGURE 3:**
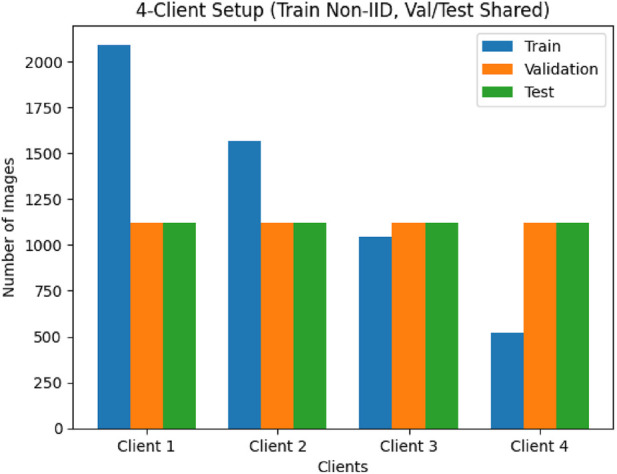
4 Client wise data split for federated Experiment (4 Clients).

There are two non-IID scenarios carried out,To provide additional variability in data distribution non-IID (Quantity skew), clients were assigned heterogeneous train/test/validation splits. When running the experiments in total 7,464 images total 5,224 images where take for training and 1,119 is take for both test and valid, with three clients (4 epochs and 4 rounds) as shown in Figure 2, Client 1 had 50% of the data as training, while Client 2 utilized 30% for training; Client 3 had a distribution where 20% was training with 15% used each for testing and validation for all three clients. Additionally, there are four clients (6 epochs and 5 rounds) as shown in Figure 3, with the following splits: Client 1 has 40% of the data as training; Client 2 utilizes 30% for training; Client 3 contains 20% of the overall allocation for training; whereas, Client 4’s shares comprise 10% training and 15% for both testing and validation for all four clients. The splits create variability in sample size and exposure while providing additional variability among client evaluations and increasing statistical heterogeneity which closely simulates decentralized autonomous vehicle perception systems in that nodes experience wide variations with respect to time and availability of data.

To Provide additional variability in label distribution (Lable skew), the use of statistical heterogeneity was applied by allocating specific dominant object classes (categories) to each client for the training phase while leaving validation and test data the same for all clients. This ensures the model can be evaluated globally in a fair manner. In the case of a three-client scenario (4 epochs and 4 rounds), training data were allocated in a way that each client had primarily seen a subset of object categories. Client 1 focused on three dominant categories (Car, Pedestrian, and Person_sitting); Client 2 had training data that consisted of two dominant categories: Cyclist and Truck; and Client 3 was given training data based upon three additional object classifications (Misc, Tram, and Van). For training data where multiple object classes exist, each class within each training image gets assigned to the class of greatest predominance; however, randomly selected sample images without any corresponding classes will still be allocated to assure adequate coverage of the training datasets. For the four-client setup (6 epochs and 5 rounds), we have made increasing variance (heterogeneity) in the label allocations made to all clients. Each client had a dominant class pair for their training and it was assigned as such: Client 1 (Car and Pedestrian); Client 2 (Cyclist and Truck); Client 3 (Misc and Tram); Client 4 (Person_sitting and Van). This will produce the availability of locally biased model representations among the clients, resulting in increased divergence of the updates.

### 3.2 Experimental setup

The research that was performed for this study was completed via the internet using Google Colab Pro (a subscription model) with NVIDIA A100 GPUs (40 GB VRAM per GPU), which provided sufficient computing resources required to train deep learning-based object detection models in a federated setting. All experiments which used the various FedAvg methods, including FedAvg; EWHFed; VINO_EffiFedAV; and the proposed DV-FedTune, were executed under the same hardware and software conditions in order to ensure comparable results and fairness to all tested methods. The experimental environment used was developed using Python, PyTorch, and the Ultralytics YOLOv12 Framework (Ultralytics, 2024) (version 8.3.159). YOLOv12 Framework is chosen for the baseline object detection architecture because it is more capable of feature extraction, has enhanced multi-scale representation learning capabilities, and optimizes inference speed, surpassing the performance of previous iterations like YOLOv8 and YOLOv10. The architecture features elegant propagation of backbone features and optimization of detection head, which can achieve more accurate localization and still maintain the real-time inference capability. The aforementioned features make YOLOv12 suitable for federated autonomous vehicles’ perception systems in context of heterogeneous non-IID data distributions. Random seeds were fixed across all experimental runs to reduce the stochastic nature of training. A pre-trained version of the YOLOv12 was employed as the global starting point for all federated experimental runs. This approach allowed for stable initialization and faster convergence of all federated models. Input image size was set to 640 × 640 pixels because this is standard size used for training the YOLO object detection model. The AdamW optimization algorithm was utilized throughout the research and used these same hyperparameters for all tested methods. To meet the architectural requirements for decentralized training, the YOLO detection head was reconfigured to support eight object classes, which correspond to the types of objects found in the KITTI dataset (Krauss, 2025). Also, all class-based layers for the detection head were excluded from aggregating into the FL model when participating clients fed data that led to label-space inconsistencies due to non-IID data partitions. Two FL configurations were assessed for scalability and for robustness regarding the number of clients and training epochs. The first configuration had three clients that provided four local training epochs (per client) over four federated training rounds. The second configuration consisted of four clients with six training epochs (per client) over five federated training rounds. In all federated training rounds, all participating clients independently trained their individual YOLOv12 models on the authority of their respective non-IID data partitions from the KITTI dataset. Upon completion of their local training, each participating client extracted and transmitted their model weights to the server for aggregation according to the chosen aggregation strategy.

### 3.3 Evaluation metrics

#### 3.3.1 Communication cost

The Data flow between client and central server in each communication rounds is measured using this metrics. To compute the total number of parameters during a communications round, it will be assumed that each parameter (i.e., model parameters) is represented by a 32-byte (4-byte) floating point number. The total number of model parameters will then be determined using the number of Filtered Dictionaries (Client Updates) (Vinoth and Sasikumar, 2025). Equation 7 provides the calculation for communication cost per round,

CommunicationCostperRound=TotalParameters×4Total CommunicationCost=SumofCommunicationCostofEachRounds

$$\text{Total Communication Cost}=\overset{R}{\underset{r=1}{\sum }}\text{Communication Cost per Round}$$ (7)

#### 3.3.2 Computational complexity

This metric computes how much processor power is needed to train a model on each of the client devices. This is computed using the product of Data N , total Parameters, and Epochs E for each of the clients. The total amount of processor power consumed per Round will use this quantity across each of the participating clients, providing some indication (total) of power is required for both processing the data and updating the weights of the model locally as shown in Equation 8 (Vinoth and Sasikumar, 2025).

ComputationalComplexityperRound=N×TotalParameters×E

$$\begin{array}{ll} & \text{Total Computational Complexity}\\ & \;=\overset{R}{\underset{r=1}{\sum }}\text{Computational Complexity}\text{per Client}\\ & \;\times \text{No of Participating Clients} \end{array}$$ (8)

#### 3.3.3 Privacy epsilon

In terms of FL, this quantifies the maximum possible information leakage about each individual data point. The extent of leakage is influenced by the total amount of data shared across client devices, how sensitive model parameters are to individual clients, and the amount of noise that is added during training to protect the local client’s privacy with respect to the training of the model calculated using Equation 9 (Vinoth and Sasikumar, 2025).

$$\text{Privacy Epsilon}\left(\epsilon \right)=\text{data sent}\times \text{sensitivity}\times \text{noise multiplier}$$ (9)

#### 3.3.4 Total Parameters

The parameter count for a FL model includes all the parameters of the entire global model, thereby defining the total size of the model. The parameter count is calculated as the sum of all elements over the model’s parameter tensors. The YOLOv12l model that we used in this study has 10,125,256 trainable parameters when trained under the KITTI 8-class configuration. For VINO_EffiFedAV and DV-FedTune, the count of communicated parameters was reduced to 2,531,648 because of excluding class-dependent detection head layers from the aggregation protocol. The total count of parameters communicated during a round of FL protocol is calculated as (shown in Equation 10) the total number of parameters in the model multiplied by the number of filtered states dicts (the clients that provided their model updates) (Vinoth and Sasikumar, 2025).

$$\text{Total Parameters}=\overset{n}{\underset{i=1}{\sum }}P_{i}\cdot \text{Num of Clients}$$ (10)

#### 3.3.5 Mean average precision (mAP)

It provides a comprehensive metric for evaluating ranking quality by computing the model’s average precision at different levels of recall using the Equation 11.

$$mAP=\frac{\sum_{i=1}^{n}\left[P_{i}\left(R_{i}\right)\cdot 4R_{i}\right]}{n}$$ (11)

#### 3.3.6 Precision

The positive prediction accuracy is represented as a percentage of the model’s total number of successful positive predictions which is calculated using Equation 12. This means that this metric shows how well the model accurately identifies appropriate cases and how trustworthy and accurate it is.

$$\text{Precision}=\frac{TP}{TP+FP}$$ (12)

#### 3.3.7 Recall

The positive prediction ratio is the number of true positives divided by the number of true and false predictions which is calculated using Equation 13. The positive prediction ratio is a way to measure a model’s capability to accurately identify similar examples.

$$\text{Recall}=\frac{TP}{TP+FN}$$ (13)

## 4 Results and discussion

**FIGURE 4:**
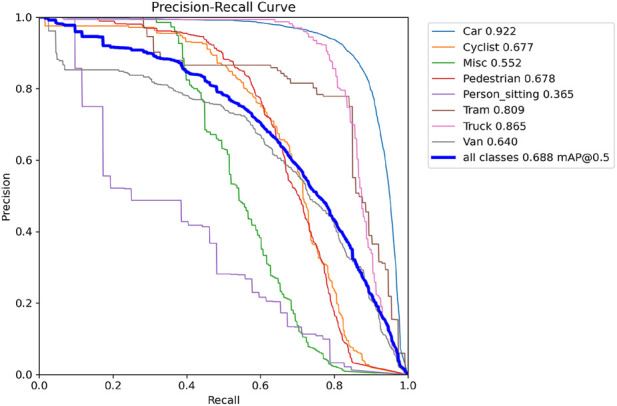
Quantity Skew: Precision Recall Curve of FedAVG (3 clients 4 epochs 4 rounds).

**FIGURE 5:**
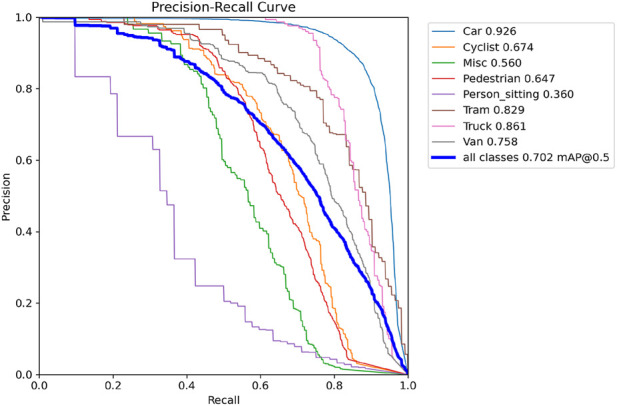
Quantity Skew: Precision Recall Curve of EWHFed (3 clients 4 epochs 4 rounds).

**FIGURE 6:**
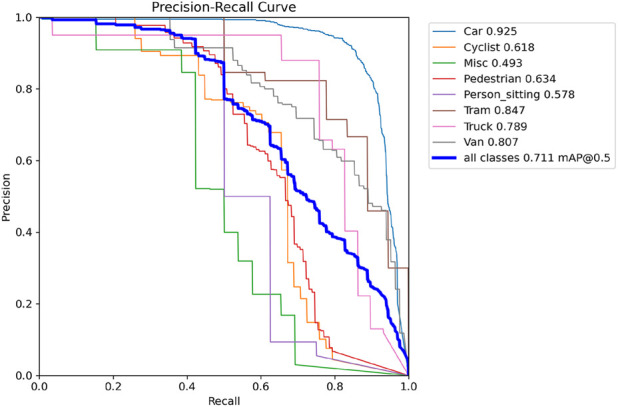
Quantity Skew: Precision Recall Curve of Vino_EffiFedAV (3 clients 4 epochs 4 rounds).

**FIGURE 7:**
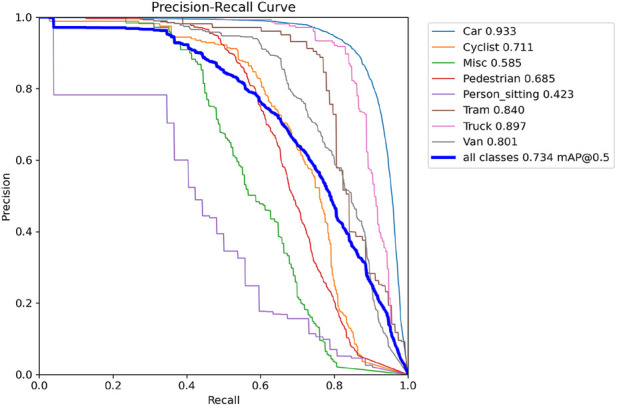
Quantity Skew: Precision Recall Curve of DV-FedTune (3 clients 4 epochs 4 rounds).

**FIGURE 8:**
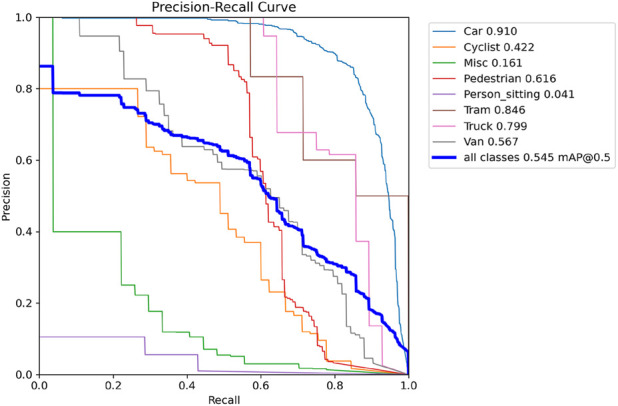
Label Skew: Precision Recall Curve of FedAVG (3 clients 4 epochs 4 rounds).

**FIGURE 9:**
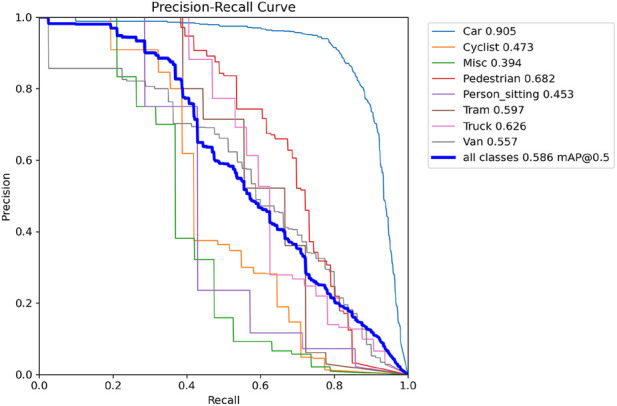
Label Skew: Precision Recall Curve of EWHFed (3 clients 4 epochs 4 rounds).

**FIGURE 10:**
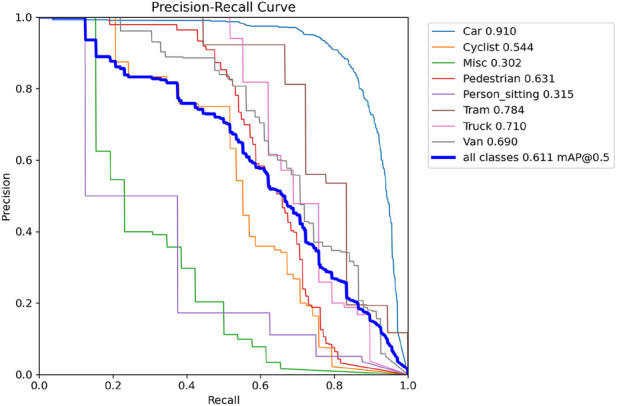
Label Skew: Precision Recall Curve of Vino_EffiFedAV (3 clients 4 epochs 4 rounds).

**FIGURE 11:**
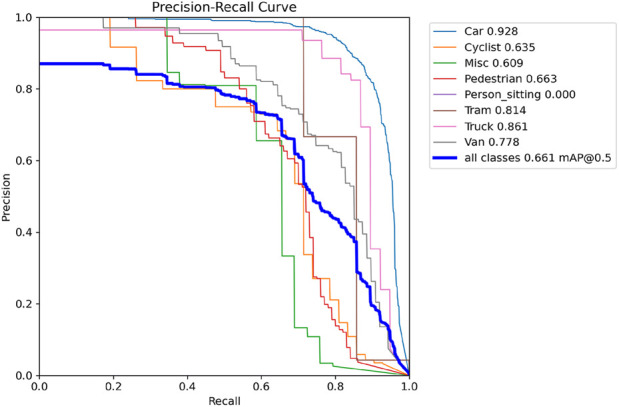
Label Skew: Precision Recall Curve of DV-FedTune (3 clients 4 epochs 4 rounds).

**FIGURE 12:**
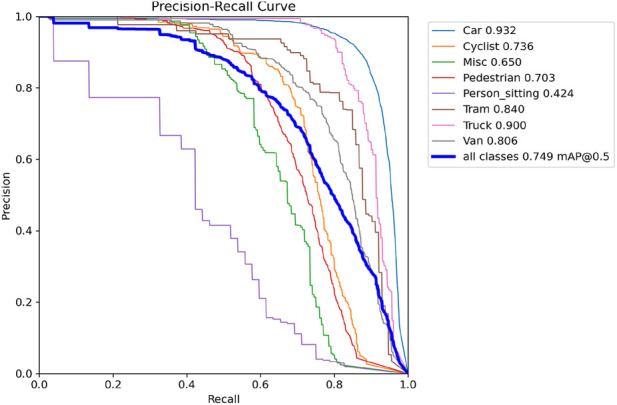
Quantity Skew: Precision Recall Curve of FedAVG (4 clients 6 epochs 5 rounds).

**FIGURE 13:**
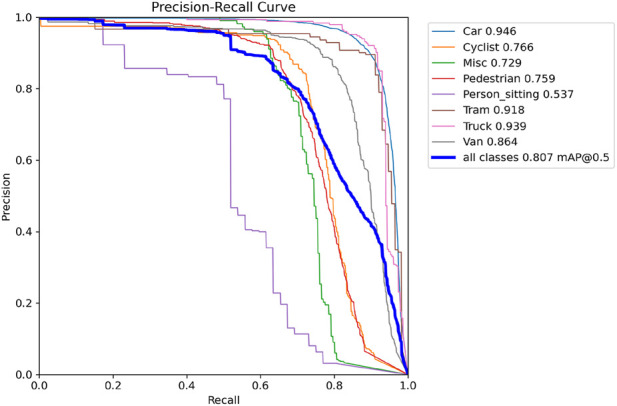
Quantity Skew: Precision Recall Curve of EWHFed (4 clients 6 epochs 5 rounds).

**FIGURE 14:**
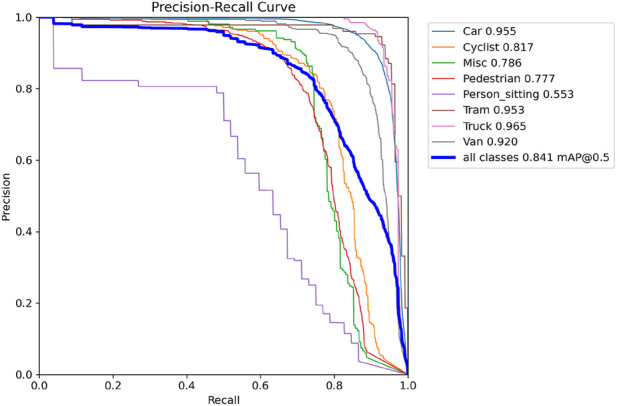
Quantity Skew: Precision Recall Curve of Vino_EffiFedAV (4 clients 6 epochs 5 rounds).

**FIGURE 15:**
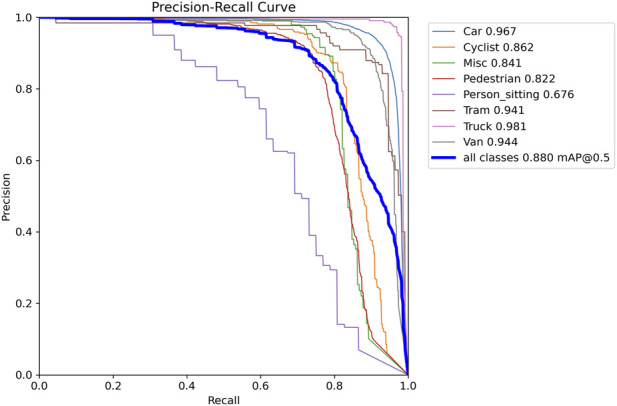
Quantity Skew: Precision Recall Curve of DV-FedTune (3 clients 4 epochs 4 rounds).

**FIGURE 16:**
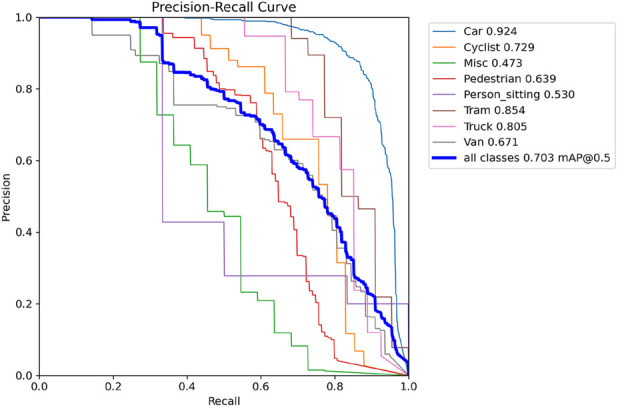
Label Skew: Precision Recall Curve of FedAVG (4 clients 6 epochs 5 rounds).

**FIGURE 17:**
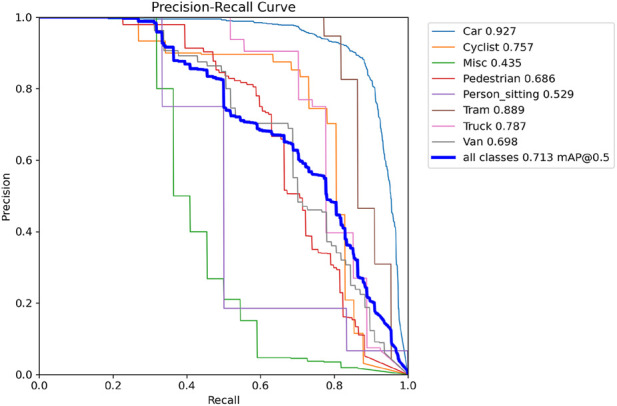
Label Skew: Precision Recall Curve of EWHFed (4 clients 6 epochs 5 rounds).

**FIGURE 18:**
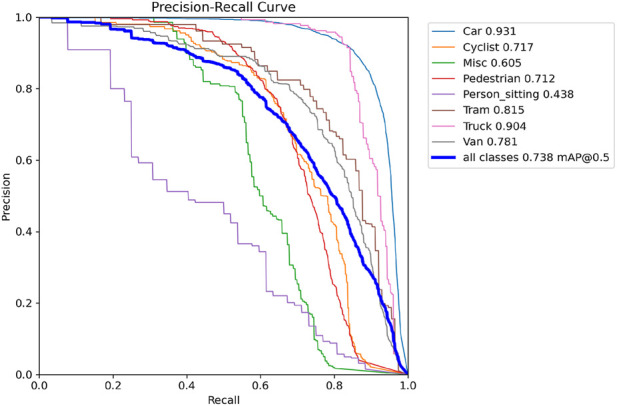
Label Skew: Precision Recall Curve of Vino_EffiFedAV (4 clients 6 epochs 5 rounds).

**FIGURE 19:**
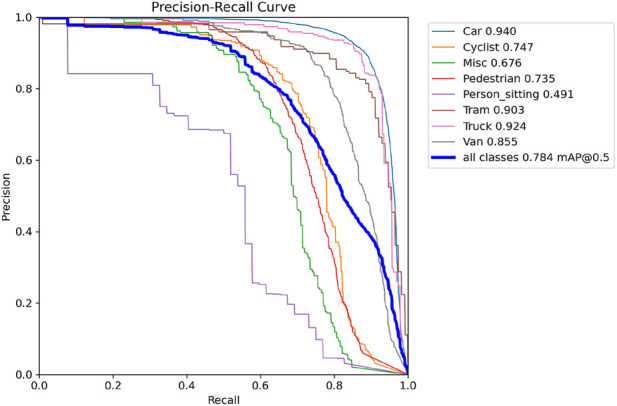
Label Skew: Precision Recall Curve of DV-FedTune (4 clients 6 epochs 5 rounds).

**FIGURE 20:**
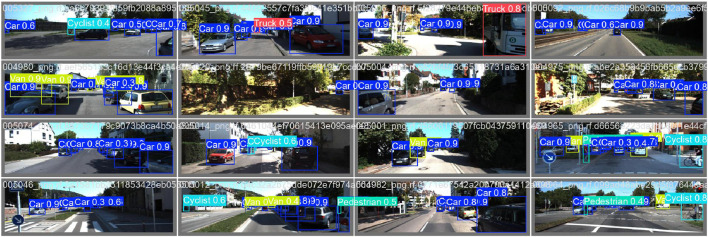
Perception results of DV-Fedtune under Quntity Skew non-IID settings (465 Configuration).

**FIGURE 21:**
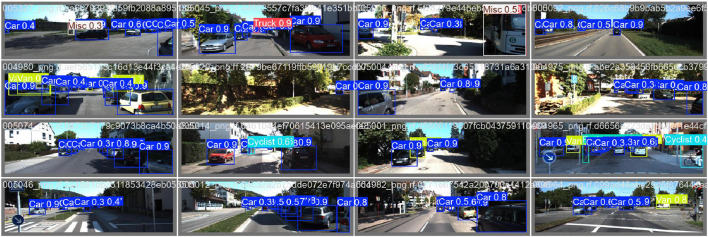
Perception results of DV-Fedtune under Label Skew non-IID settings (465 Configuration).

Experimental evaluations of DV-FedTune have been conducted that assess the effectiveness of DV-FedTune against FedAvg, EWHFed, and VINO_EffiFedAV under two sets of non-IID conditions, 3 clients and 4 clients with quantity skew and label skew. In each of the three client configurations, the experimental results show the highest average mAP values for DV-FedTune compared to all other methods. The mAP@0.5 values for quantity skew (as shown in Table 1 are FedAvg 68.8% (Figure 4), EWHFed 70.2% (Figure 5), VINO_EffiFedAV 71.1% (Figure 6), and DV-FedTune 73.4% (Figure 7), showing that the use of dynamic variance-aware weighting effectively mitigated the effects of heterogeneous data volume distributions. For label skew in the 344 configuration, the average mAP values are FedAvg 54.5% (Figure 8), EWHFed 58.6% (Figure 9), VINO_EffiFedAV 61.1% (Figure 10), and DV-FedTune 66.1% (Figure 11), highlighting the ability of divergence-aware and loss-guided aggregations to reduce the degree of class imbalance as a result of the use of static aggregation methods. However, in the 4 client configuration, the mAP values for each aggregation technique improved because more clients and more training rounds were used; however, DV-FedTune continued to perform better compared to other methods. With respect to quantity skew, FedAvg 74.9% (Figure 12), EWHFed 80.7% (Figure 13), VINO_EffiFedAV 84.1% (Figure 14), and DV-FedTune 88.0% (Figure 15). In contrast, the mAP values for the same methods for label skew are (as shown in Table 2) FedAvg 70.3% (Figure 16), EWHFed 71.3% (Figure 17), VINO_EffiFedAV 73.8% Figure 18, and DV-FedTune 78.4% (Figure 19). Based on the results of the experiments, DV-FedTune can be viewed as an effective FL method that is better able to balance, scale, and perform perception tasks (Shown in Figures 20, 21) for an autonomous vehicle when using either configuration 344 or 465 in both non-IID scenarios.

**TABLE 1:** Comparative detection performance of FL methods on the KITTI dataset under non-IID settings (344 configuration).

Aggregation technique	Quantity skew (344)			Label skew (344)		
	mAP@0.5 (%)	Precision (%)	Recall (%)	mAP@0.5 (%)	Precision (%)	Recall (%)
FedAvg	68.8	99.5	89	54.5	99.7	78
EWHFed	70.2	97.3	87	58.6	93.4	81
Vino_EffiFedAV	71.1	99.0	83	61.1	95.8	80
Dv-Fed-Tune (Ours)	**73.4**	95.2	87	**66.1**	99.0	77

Bold values indicate the highest mAP@0.5 score achieved.

**TABLE 2:** Comparative detection performance of FL methods on the KITTI dataset under non-IID settings (465 configuration).

Aggregation technique	Quantity skew (465)			Label skew (465)		
	mAP@0.5 (%)	Precision (%)	Recall (%)	mAP@0.5 (%)	Precision (%)	Recall (%)
FedAvg	74.9	99.0	74.9	70.3	93.5	88
EWHFed	80.7	99.0	89	71.3	94.8	88
Vino_EffiFedAV	84.1	97.4	92	73.8	99.6	89
Dv-FedTune (Ours)	**88.0**	99.2	93	**78.4**	95.4	90

Bold values indicate the highest mAP@0.5 score achieved.

Communication cost analysis examines how scalable different feed aggregation strategies may be in the different configurations. As in 344 configuration, both FedAvg and EWHFed incur a constant cost for communication overhead of 1.215 in each round because full model is sent between all clients, while DV-FedTune is much lower in cost than either at 0.405, or approximately 66.7% less than FedAvg.When comparing the configurations of 465, both FedAvg and EWHFed will exhibit higher communication costs at 1.215 than they did at 344; this is due to both clients sending more data (increased client subscriptions) and training many more rounds. Although VINO_EffiFedAV will have a communication cost of 2.548 at this configuration, DV-FedTune will be less than that cost as well at 0.405; its ability to have low communication costs reflects not only that it has been designed for scalability but also that it has made efficient use of its parameters in these configurations (as shown in Table 3), because it has said to have been created with divergence aware weighting in both configurations.

**TABLE 3:** Communication cost per round under quantity skew and label skew settings.

Aggregation technique	Q (344)		Q (465)		L (344)		L (465)	
	Raw	GB	Raw	GB	Raw	GB	Raw	GB
FedAvg	303,762,768	1.215	405,017,024	1.620	303,762,768	1.215	405,017,024	1.620
EWHFed	102,993,056	0.412	102,993,056	0.412	102,993,056	0.412	102,993,056	0.412
Vino_EffiFedAV	637,114,777	2.548	849,482,875	3.398	607,600,000	2.430	810,130,000	3.241
Dv-FedTune (Ours)	101,266,592	0.405	101,266,592	0.405	101,266,592	0.405	101,266,592	0.405

The Table 4 show the effectiveness of using DV-FedTune with regard to computational complexity. The 344 configuration of FedAvg shows the highest computational requirement of all models. The computational demand for FedAvg is 5492.44. FedAvg has the largest amount of processing power needed, but EWHFed provides some reduction in computational variance due to smoothing of temporal data, but it still operates on complete client data and thereby shows similar variability in overhead. The VINO_EffiFedAV requires processing demand of 911.29 as the low value of weight in state will cause less total computational requirement, and DV-FedTune reduces that requirement to 303.80 resulting in a 94% reduction in computational demand from FedAvg and a significant improvement from VINO_EffiFedAV.The 465 configuration of FedAvg requires 2,430 computational requirements, EWHFed has similar requirements to FedAvg due to the use of full updating mode. The VINO_EffiFedAV requires 1,098 computational requirements and DV-FedTune has the least of the four models at 607 computational requirements. Although there are still increases in total computational costs as you scale the system down, DV-FedTune continually maintains an overall reduced processing footprint compared to the other three baseline models which shows that the use of adaptive variance aware weighting to limit unnecessary overhead of optimization is effective.

**TABLE 4:** Computational complexity (GFLOPs per round) under quantity skew and label skew settings.

Aggregation technique	Quantity (344)	Quantity (465)	Label (344)	Label (465)
FedAvg	5492.44	2430.10	911.29	2430.10
EWHFed	308.98	617.96	308.98	617.96
Vino_EffiFedAV	911.29	1098.49	52380.00	104760.00
Dv-FedTune (Ours)	303.80	607.60	303.80	607.60

The analysis evaluates the extent to which different aggregation methods preserve privacy using the privacy epsilon metric. In the 344 configuration of the analysis, FedAvg has a significant reduction in privacy epsilon from 119.99 to 30 as a result of repeated transmissions of the full set of parameters from multiple clients which resulted in cumulative exposure to performance and privacy degradation. Similarly, EWHFed has an increase in cumulative privacy exposure because it has also transmitted a complete set of parameters. In comparison (Shown in Table 5), VINO_EffiFedAV accomplishes a significant reduction to 3.80 in the final privacy epsilon value by limiting the number of original updates transmitted. Finally, DV-FedTune achieves the lowest final value of 1.26 in privacy epsilon with an overall decrease from 20.25. In the 465 configuration of this analysis, FedAvg exhibits an overall reduction in privacy epsilon value from 199.98 to 40; this indicates a high degree of cumulative exposure to privacy loss and data degradation from the large number of repetitions required to perform training in these types of experiments. EWHFed shows a similar decrease trend as a result of transmitting the full parameter set per client as well. Unlike both VINO_EffiFedAV (stabilization of privacy epsilon at 3.24) and DV-FedTune (final reduction of privacy epsilon to 0.81), there were significant differences among the three methods concerning privacy leakage. Overall, these results show that reductions in saturation times and increases in stabilization percentages can improve the level of control over the disclosure of sensitive data through an adaptive weighting system of client contribution during an experiment.

**TABLE 5:** Privacy epsilon (ϵ) per communication round under quantity skew and label skew settings.

Technique	Quantity (344)	Quantity (465)	Label (344)	Label (465)
FedAvg	60.75	81.00	60.75	81.00
EWHFed	20.60	20.60	20.60	20.60
Vino_EffiFedAV	119.99	159.98	5321.00	199.98
Dv-FedTune (Ours)	20.25	20.25	20.25	20.25

**FIGURE 22:**
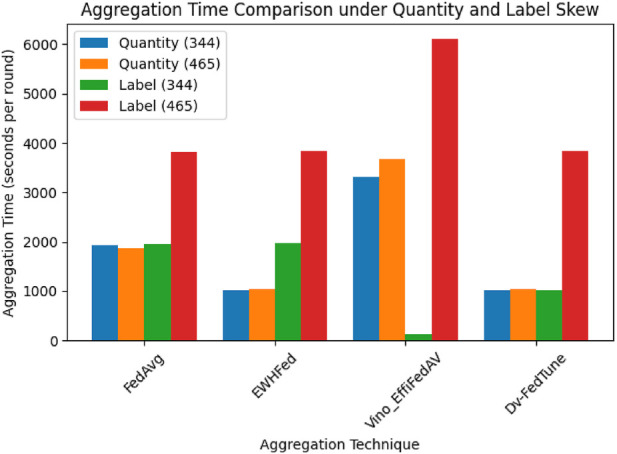
Aggregation Time comparison under Quantity and Label Skew.

The elapsed time for aggregation is an indicator of the stability of convergence across disparate distributions of data (Shown in Table 6). FedAvg has the most burdened aggregation due to both the increasing communication and computational requirements when using the configurations mentioned (particularly the 465 configuration) in which communication costs have a maximum value of 0.850 and computation costs have maximum values of 2,430. EWHFed improves smoothness of convergence by applying a historical weight, exponentially; however, the fact that EWHFed does not directly address both the divergence of updates nor the statistical variance also results in the aggregation time being impacted by misaligned updates from client(s). With the use of a magnitude based filtering approach, VINO_EffiFedAV provides some measure of stabilization; however, its static client selection process restricts adaptability. On the other hand, DV-FedTune provides consistent aggregation efficiency (as shown in Figure 22) for both configurations with an average communication cost of 0.101 and computation costs of 303.80 and 607, respectively, for configuration 344 and configuration 465. By using a round-adaptive multi-factor weighting method, oscillations due to divergent updates are minimized, thus leading to smoother global convergence.

**TABLE 6:** Aggregation time (seconds per round) under quantity skew and label skew settings.

Aggregation technique	Quantity (344)	Quantity (465)	Label (344)	Label (465)
FedAvg	1927.98	1860.13	1940.63	3813.72
EWHFed	1014.60	1045.18	1969.20	3833.16
Vino_EffiFedAV	3306.00	3671.00	119.99	6104.00
Dv-FedTune (Ours)	1014.73	1038.99	1025.93	3844.00

With 10,125,256 trainable parameters, the YOLOv12 model reveals a considerable communication overhead related to parameter transfers under both quantity skew (344 samples) and label skew (465 samples). The baseline FedAvg algorithm transmits 75,940,692 parameters per round when faced with quantity skew, increasing to 101,254,256 with increased sample numbers (shown in Table 7). In contrast, the EWHFed and Dv-FedTune methods markedly reduce this transmission to ranges between 25,748,264 and 25,316,648 parameters, thereby enhancing communication efficiency. However, the Vino_EffiFedAV method yields significantly higher transmission figures, reaching 637,114,777 parameters. FedAvg transmits 101,254,256 parameters under label skew conditions, but EWHFed and Dv-FedTune continue to transmit around 25 million parameters. Interestingly, Dv-FedTune provides better performance metrics while minimising parameter transmission, suggesting that decreased transmission does not affect detection accuracy.

**TABLE 7:** Total number of parameters transmitted per round under quantity skew and label skew settings.

Technique	Quantity (344)	Quantity (465)	Label (344)	Label (465)
FedAvg	75,940,692	101,254,256	75,940,692	101,254,256
EWHFed	25,748,264	25,748,264	25,748,264	25,748,264
Vino_EffiFedAV	25,316,648	25,316,648	25,316,648	25,316,648
Dv-FedTune (Ours)	25,316,648	25,316,648	25,316,648	25,316,648

The statistical patterns which were measured during both experimental conditions show that DV-FedTune delivers a superior federated optimization solution which operates with greater strength and flexibility. The system achieves its optimal performance through dynamic client contribution control which improves both efficiency and protection of private information when handling non-IID data distributions, thus making it suitable for extensive autonomous driving perception systems.

## 5 Conclusion

The DV-FedTune, a federated aggregate algorithm that adapts dynamically according to the variance of input. The purpose of this algorithm is to assist in the FL of AVs collecting data and designing object detection systems. An alternative method was implemented using the YOLOv12 object detection algorithm, considering multiple federated configurations on the KITTI dataset. The results of several experiments have shown the superiority of DV-FedTune with respect to its capability to aggregate client updates compared to FedAvg, EWHFed and VINO_EffiFedAV, as measured by detection accuracy, communication costs, aggregation times, and privacy-epsilon computational complexity. Furthermore, DV-FedTune achieved greater mAP@0.5, precision, and recall, while having a lower privacy epsilon and a smaller aggregation overhead than the others. Accordingly, the use of DV-FedTune developed improved stability and efficiency in machine learning through heterogeneous data distributions. The method that has been proposed also demonstrated a reliable computational workload for complexity of parameter stability during rounds of training, making it ideal for the use large-scale federated deployments. Overall, DV-FedTune provides an efficient scalable solution to privacy preserving collaborative learning for autonomous driving environments. As well as providing balanced performance, efficiency and privacy, this new framework will help to advance the current state of federated object detection and provide an excellent basis for intelligent vehicular perception systems to be developed for distributed real-world applications in the future.

## 6 Limitations and future work

Although DV-FedTune shows promise in its use, there are still numerous limitations that require further research. Firstly, the methodology for aggregating the data has primarily been tested with respect to the KITTI dataset, which uses a fixed object detection structure (YOLOv12). The KITTI dataset provides an appropriate comparative test for systems meant for AVs; however, it is structured in urban settings and therefore may not adequately reflect extreme weather events, as well as the type of rural settings, or non-daytime settings that real vehicles are subjected to. To improve generalizations of DV-FedTune to very different real-world driving conditions, the use of different datasets that consist of large amounts of heterogeneous data is advised. Secondly, although the algorithm has improved its robustness in handling much of the non-IID distribution of client data during the client side of the aggregation process, the implementation assumes that all clients will be present, and none of them will be subject to dropouts, tardiness, or intermittent network connectivity issues-all of which are typically seen among client devices within real world vehicle networks. Privacy evaluations will be conducted using estimated privacy epsilon, with no formal differential privacy guarantees, allowing for potential improvements to privacy-preserving mechanisms in the future. Future work on extending DV-FedTune capabilities to other multi-modal perception tasks such as LiDAR and radar combined with vision models will be done, as well as investigating ways to include formal differential privacy or secure aggregation protocols as an avenue for improving privacy guarantees. Adaptive client selection and robust aggregation techniques to resist attacks from malicious or unreliable parties will be evaluated as well. Finally, real world automotive testbeds and edge-deployment studies will be conducted to evaluate scalability and real-time usability of the methods.

## Data Availability

The raw data supporting the conclusions of this article will be made available by the authors, without undue reservation.
